# Lifting as we climb: Experiences and recommendations from women in neural engineering

**DOI:** 10.3389/fnins.2023.1104419

**Published:** 2023-03-09

**Authors:** Maria K. Jantz, Jennifer Mak, Ashley N. Dalrymple, Juhi Farooqui, Erinn M. Grigsby, Angelica J. Herrera, Elvira Pirondini, Jennifer L. Collinger

**Affiliations:** ^1^Rehab Neural Engineering Labs, University of Pittsburgh, Pittsburgh, PA, United States; ^2^Department of Bioengineering, University of Pittsburgh, Pittsburgh, PA, United States; ^3^Center for Neural Basis of Cognition, Pittsburgh, PA, United States; ^4^Department of Mechanical Engineering, Carnegie Mellon University, Pittsburgh, PA, United States; ^5^Neuroscience Institute, Carnegie Mellon University, Pittsburgh, PA, United States; ^6^Department of Physical Medicine and Rehabilitation, University of Pittsburgh, Pittsburgh, PA, United States; ^7^Department of Biomedical Engineering, Carnegie Mellon University, Pittsburgh, PA, United States

**Keywords:** neural engineering, academic careers, gender representation, equity, inclusion and diversity, bias, STEM, academia

## Abstract

Neural engineering is an emerging and multidisciplinary field in which engineering approaches are applied to neuroscience problems. Women are underrepresented in engineering fields, and indeed in science, technology, engineering, and mathematics (STEM) fields generally. Underrepresentation of women is particularly notable at later academic career stages, suggesting that even though women are interested in the field, barriers exist that ultimately cause them to leave. Here, we investigate many of the obstacles to women’s success in the field of neural engineering and provide recommendations and materials to overcome them. We conducted a review of the literature from the past 15 years regarding the experiences of women in academic careers, as well as reports on the number of women in fields closely related to neural engineering from the National Science Foundation (NSF) and the American Society for Engineering Education (ASEE). Additionally, we interviewed six women in neural engineering who are involved in initiatives and outreach concerning the inclusion and experiences of women in engineering. Throughout the literature and interviews, we identified common themes spanning the role of identity and confidence, professional relationships, career-related hurdles, and personal and professional expectations. We explore each of these themes in detail and provide resources to support the growth of women as they climb within the field of neural engineering.

## 1. Introduction

**FIGURE 1:**
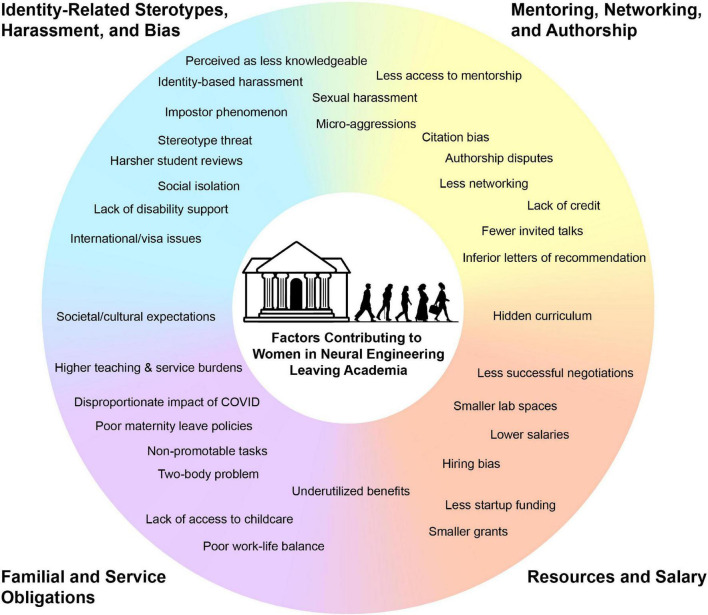
Factors contributing to women in neural engineering leaving academia. These factors were noted by our interviewees and found throughout literature from fields related to neural engineering. Some factors may be applicable to anyone leaving academia, but many are uniquely experienced by women. They are categorized into: Identity-related stereotypes, harassment, and bias; mentoring, networking, and authorship; resources and salary; familial and service obligations. Some factors overlapped categories. Two-body problem is defined as the difficulty of finding jobs in the same geographic region for couples in which one or both partners are seeking academic positions.

Despite years of research calling attention to the issue, gender imbalances persist in science, technology, engineering, and mathematics (STEM) fields (Chesler et al., 2010; Hussénius, 2020; Machlovi et al., 2021). For women who set out on STEM career paths, these imbalances are evident in the expectations and assumptions directed at them in their workplaces. For example, women are often perceived as less competent than their male peers (Moss-Racusin et al., 2012) and are asked to take on a greater number of service tasks that do not contribute to their career advancement (Casad et al., 2021). These experiences create work environments that can feel isolating and inhospitable, leading women to take on additional work trying to address the inequities they experience, and in many cases leading them to leave the field entirely (Figure 1). Indeed, there is a steady attrition of women’s representation in STEM fields at every stage in the academic career path (National Science Foundation [NSF], 2017a).

While obstacles to women’s achievement in STEM fields are well documented, little has been written about gender representation and women’s experiences in neural engineering. Neural engineering is an emerging field in which quantitative methods are applied to solve neuroscience problems and develop rehabilitative technology for neural disorders (Durand, 2006). This paper provides an overview of many of the obstacles women and gender minorities may encounter in pursuit of careers in neural engineering, presented through the lens of the experiences of several women in the field. In order to investigate these barriers, and highlight some efforts to address them, we share the perspectives of several United States-based women who have been active in creating and leading initiatives to support women and other historically marginalized identities in the field. We have sought to amplify the voices of women at various stages in their careers, and with diverse identities and life experiences (e.g., historically marginalized racial backgrounds, immigration status, etc.). Through the experiences and perspectives shared by these women, we seek to shed some light on the state of the neural engineering field and situate it within the broader STEM landscape. To contextualize the narratives shared by our interviewees, we provide a review of literature on gender representation and gendered experiences in STEM, specifically directed by the themes that emerged from our interviews. We also identify key strategies that our interviewees have developed to address some of the systemic barriers they have experienced and conclude with a list of resources and materials to support women in neural engineering at various career stages. Furthermore, although gender minority experiences remain underrepresented in the literature and our interviewees were all cisgender women, it is worth noting that people who are trans, non-binary, or part of other gender minorities may experience many of the same obstacles as cisgender women, in addition to further discrimination related to gender identity.

Many of the experiences we highlight in this paper align with broader trends in STEM fields and academia. While the structural inequities and barriers discussed in this paper are not unique to neural engineering, they represent a critical call to action. As a young and rapidly growing field, neural engineering has a unique opportunity to reckon with these systemic barriers early. Doing so will expand the potential of this field to generate vibrant, meaningful, and impactful science that is both driven by and responsive to diverse needs and perspectives.

## 2. Approach

We performed a literature search of papers discussing gendered experiences in neural engineering academic careers. Because neural engineering is a relatively young field and is very interdisciplinary, there are few field-specific statistics. Therefore, we prioritized literature from engineering, neuroscience, and biological sciences, published primarily within the last 15 years. Papers published prior to 2007 were only cited if we were analyzing changes over time or if more recent citations were lacking. We also used the Balanced Citer tool created by Dr. Dani Bassett’s laboratory to check our reference lists for gender equity and appropriate representation (Dworkin et al., 2020). This tool uses datasets of baby names and social media profiles to evaluate the first names of authors and identify their likely gender, with “man” or “woman” assigned to names that have a greater than 70% probability of belonging to that gender.

Notably, data regarding the experiences of scientists who are non-binary, trans, or genderqueer are scarce. Most large-scale datasets, including the Survey of Earned Doctorates, American Society for Engineering Education (ASEE) reports, and National Science Foundation (NSF) reports contain only male and female identities, which further means that any paper relying on these datasets cannot investigate important questions regarding gender minorities. Cisgender scientists ranging from undergraduates to professionals are more likely than trans or gender non-conforming scientists to remain in STEM fields (Cech and Waidzunas, 2021; Maloy et al., 2022), and to rectify this inequality it is essential that future datasets collect more accurate gender identities. Many studies cited in this paper do not disambiguate trans and gender non-conforming individuals from cisgender men and women; where possible we have provided data that explicitly describes those experiences.

Using historical data and reports provided by the ASEE and the NSF, we generated time-series plots showing the proportion of individuals that identified as women in biomedical, mechanical, and electrical engineering at the bachelors, doctorate, and faculty levels over time, from 1983 to 2021. Each of these fields are relevant to neural engineering; biomedical engineering is the most similar and neural engineering is often considered one of its subfields. For each field of engineering, we performed a linear regression to obtain the equation for the line of best fit. Using that equation, we calculated the year in which men and women are expected to be in equal proportion at the faculty level at the current rate. We also compiled historical data from the NSF for biological sciences at the bachelors, masters, doctorate, post-doctorate, assistant professor, associate professor, and full professor levels from 1979 to 2020. We were unable to compile historical data for neuroscience because reporting of biological sciences subfields was sparse and inconsistent.

We interviewed six United States-based women (Table 1) who have started or led initiatives supporting women and/or minoritized people in neural engineering, neuroscience, and STEM broadly. These interviews took place between May and September of 2022. Many of their initiatives focus on intersectional advocacy, supporting people who are historically marginalized based on gender as well as race and ethnicity, disability, and queerness. The interviewees represent different career stages, ranging from graduate students to faculty members and industry leaders. They also represent many different experiences and backgrounds that intersect with gender, including racial and ethnic identity (Black, Hispanic, White, Chinese), immigration status (born in the US, first- or second-generation immigrant), sexuality, disability status, and parenthood status.

**TABLE 1:** Interviewees and their appointments and affiliations at the time of the interview.

Interviewee	Appointment	Affiliation
Dr. Lena Ting	Professor	Wallace H. Coulter Department of Biomedical Engineering, Emory University and Georgia Institute of Technology, Atlanta, GA, United States
Dr. Maribel Vazquez	Professor	Department of Biomedical Engineering, Rutgers University, New Brunswick, NJ, United States
Dr. Amy Orsborn	Assistant Professor	Department of Electrical and Computer Engineering, University of Washington, Seattle, WA, United States
Dr. Elisa Castagnola	Research Assistant Professor	Department of Bioengineering, Swanson School of Engineering, University of Pittsburgh, Pittsburgh, PA, United States
Lietsel Jones	Ph.D. Student	Burnett School of Biomedical Sciences, University of Central Florida, Orlando, FL, United States
Dr. Erika Ross	Industry, Director	Global Clinical & Applied Research, Abbott Neuromodulation, Austin, TX, United States

Professor Lena Ting is the McCamish Foundation Distinguished Chair in Biomedical Engineering at Emory University and the Georgia Institute of Technology. She advocates for women in academia through public talks and shares resources and insights online about navigating academia and building a successful research program. Professor Maribel Vazquez, Department of Biomedical Engineering, Rutgers University, is a Director for Diverse Scholar Engagement at Rutgers University and Vice President At-Large for the American Institute for Medical and Biological Engineering (AIMBE). In those roles, she works to make neural engineering a more inclusive academic community by amplifying the work of other women in neural engineering. Professor Amy Orsborn, Department of Electrical and Computer Engineering, University of Washington, is one of the founding members of the Women in Neural Engineering (WINE) Forum, which is a network of women in neural engineering at all stages of their academic career. The WINE forum hosts socials and networking opportunities, and also provides personalized career mentorship. Professor Orsborn also leads professional development seminars in her lab group and provides resources for women and people with disabilities on Twitter. Professor Elisa Castagnola, who at the time of the interview was in the Department of Bioengineering, University of Pittsburgh, works on Inequality Stories In STEM, a website created by Professor Dawn Taylor, where women can submit their anonymous anecdotes of incidents they have experienced. She has presented these stories at several conferences to raise awareness of the inequalities that women in STEM face and the changes that are needed to improve equality. Lietsel Jones was a graduate student in the Burnett School of Biomedical Sciences, University of Central Florida at the time of the interview. She is a co-founder of Black in Neuro, an organization that celebrates the work of Black neuroscientists, holds workshops and networking sessions, and creates a sense of community for Black neuroscientists all around the world. Dr. Erika Ross was Director of Global Clinical and Applied Research at Abbott Neuromodulation and serves as the chair of Women in Engineering & Diversity and Inclusion for the IEEE Engineering in Medicine and Biology Society. As a mentor, she has advocated for women in the neurotech industry by providing women with opportunities to lead and be successful. Three interviewees have changed appointments and affiliations since the interviews: Dr. Castagnola is now an Assistant Professor in Biomedical Engineering at Louisiana Tech University, and Ms. Jones has left academia, and Dr. Erika Ross is now Vice President, Global Clinical & Regulatory at ONWARD.

Our interview questions were designed to explore these individuals’ experiences in the field of neural engineering, as well as the initiatives they created and their motivations for creating them (see Supplementary material for a list of the questions). Interviews were transcribed with either the Zoom Meetings save-to-Cloud function (Zoom Video Communications, San Jose, CA, USA) or Microsoft Word Online (Microsoft 365, Redmond, WA, USA) to facilitate authorial review. Personal experiences from the interviewees were used to illustrate and supplement trends and themes identified in the literature review. Finally, we curated a set of informational materials and strategies to address inequity in neural engineering and STEM in general. We compiled these materials and strategies based on our interviews, the papers discussed in this manuscript, and resources that we have found helpful throughout our own careers, and have shared them in the final section of this paper.

## 3. Barriers to women’s academic careers

We identified several themes our interviews, which are further borne out in the literature. Themes leading to attrition of women and gender minorities in the field include the influence of societal pressures, unequal access to networks and mentorship, social hostilities, disproportionate expectations for teaching and service, and inadequate parental and disability support. These challenges have led to the creation of initiatives to make academia a more hospitable environment for women and gender minorities. The initiatives created by our interviewees include both formal organizations and informal mentorship and support. Through these initiatives, these women have greatly improved the neural engineering ecosystem by providing resources including workshops, networking opportunities, and support structures at their own institutions and within the broader scientific community. However, there are several areas in which greater support is needed to retain women in neural engineering. In the following sections, we enumerate these areas identified by our interviewees and literature reviews.

### 3.1. Representation of women across career stages and in authorship positions

**FIGURE 2:**
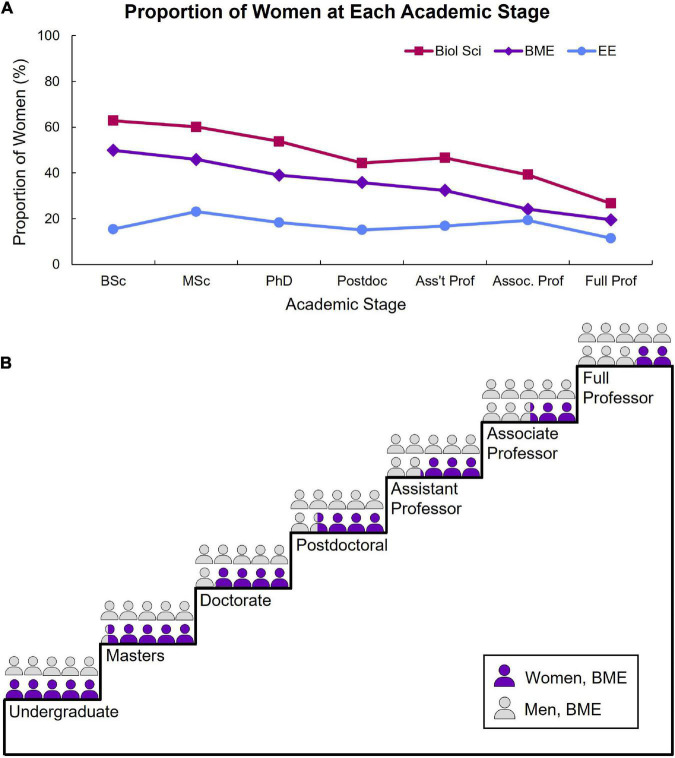
The attrition of women from fields related to neural engineering. **(A)** Graph shows the proportion of women in each academic stage for three fields related to neural engineering: Biological sciences (Biol Sci), biomedical engineering (BME), and electrical engineering (EE). Data were collected from annual and biannual reports from the American Society for Engineering Education [ASEE] (2020) and National Science Foundation [NSF] (2021). **(B)** Graphic highlights the attrition of women in biomedical engineering across each academic stage, represented by a climbing staircase.

Biomedical engineering, which is the engineering field most analogous to neural engineering, boasts a relatively higher proportion of women at the undergraduate level compared with other engineering fields (American Society for Engineering Education [ASEE], 2021b). However, like other STEM fields, biomedical engineering suffers from a failure to retain women at advanced academic stages. Data from ASEE reports in 2019–2020 (American Society for Engineering Education [ASEE], 2021a) and NSF reports in 2018–2019 (National Science Foundation [NSF], 2021) show a linear decline in the proportion of women at each academic stage, ranging from 49.9% of students graduating with a bachelor’s degree being women, to only 19.5% of full professors (Figures 2A, B). Biological sciences, which includes neuroscience, has a higher proportion of women than biomedical engineering across all academic stages, but also shows a linear decline to advanced career stages (Figure 2A). Electrical engineering, which also intersects with neural engineering research, has a consistently low proportion of women across all academic stages (Figure 2A). Electrical and biomedical engineering have similar proportions of women at the associate and full professor stages.

**FIGURE 3:**
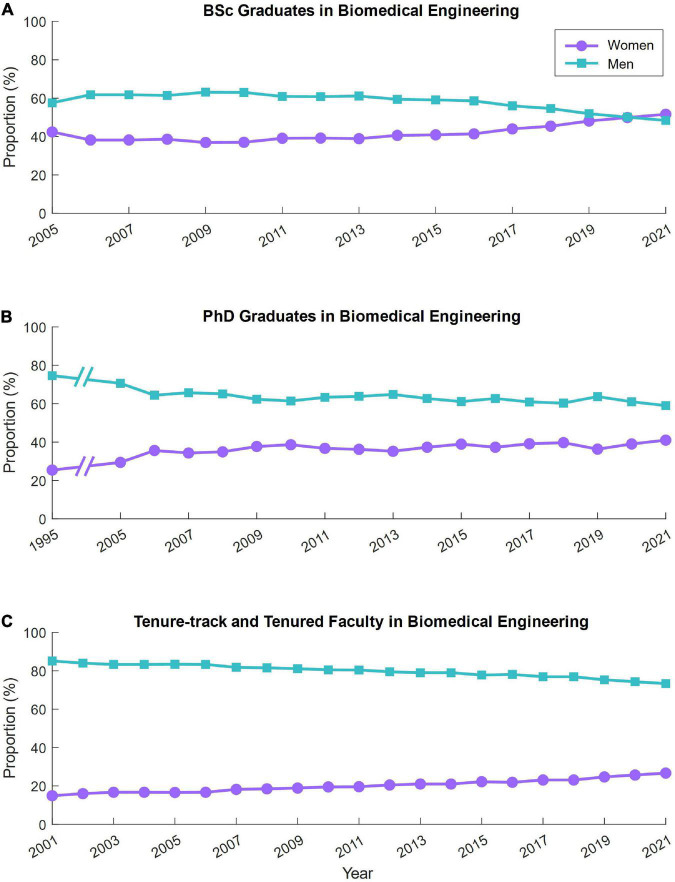
Proportion of women in biomedical engineering over time. The proportion of women in biomedical engineering graduating with a bachelor’s degree **(A)**, doctorate degree **(B)**, or who are faculty **(C)** over time. Data were collected from annual and biannual reports from the American Society for Engineering Education and the National Science Foundation. The dashed lines indicate a large gap in data availability.

Over time, there has been a relatively high proportion of women graduating with a bachelor’s degree in biomedical engineering, consistently between approximately 40–50% since 2005 (Figure 3A), with more women than men (51.6%) graduating with bachelor’s degrees in 2021. The proportion of women graduating with a Ph.D. in biomedical engineering has also been consistently high relative to other engineering fields (American Society for Engineering Education [ASEE], 2021b), with about 30–40% of graduates being women since 2005, which increased from 25.4% in 1995 (Figure 3B). Unfortunately, the proportion of women faculty in biomedical engineering is low (15–27% between 2001 and 2021; Figure 3C), demonstrating a lack of advancement of women in academia in biomedical engineering. At the current rate of increase in the proportion of women faculty in biomedical engineering, as determined by a linear regression for this time-series data, the field will reach parity (50% women faculty) in the year 2067 (*R*^2^ = 97.6%). At the current rate, mechanical engineering will reach parity in the year 2096 (*R*^2^ = 99.0%; Supplementary Figure 1) and electrical engineering in the year 2106 (*R*^2^ = 97.3%; Supplementary Figure 2). Because the proportion of women undergraduates in mechanical and electrical engineering have been consistently low for decades, one might argue that gender parity is not achievable in these fields due to disinterest. This is a multifaceted issue that likely involves stereotypes that are learned at early ages as well as micro- or macro-aggressions experienced by the few women in those fields, which we describe in detail in the following section. More needs to be done to extinguish these stereotypes and make all engineering fields a welcoming environment to women and gender minorities, otherwise we risk neural engineering, like these related fields, not reaching gender parity until the latter half of this century.

Although biological sciences is near parity at career stages prior to associate professorship (Supplementary Figures 3C–E), only 20.7–26.7% of full professors have been women since 2003 (Supplementary Figure 3F). Undergraduates reached gender parity in 1989, and doctorates in 2008 (Supplementary Figures 3A, B), but gender parity has not been reached at the post-doctorate stage, or any of the professor levels. This suggests that a ceiling is preventing women from reaching the highest level in academia, a sentiment that was noted by several of our interviewees. Fields such as biomedical engineering and biological sciences that have had 50% or more women enrolled in undergraduate studies for several years should have already achieved gender parity at the doctorate, post-doctorate, and early faculty levels. Because of the high proportion of women at the undergraduate level, these fields are at risk of becoming complacent, allowing remaining inequities to go unaddressed. This disparity at later career stages is often attributed to demographic inertia, that is, the fact that older generations have a higher proportion of men and they simply have not reached retirement. However, that effect does not fully account for the disparity, suggesting that women encounter additional barriers to career success (Shaw and Stanton, 2012). These barriers must be addressed actively to reach gender equity in the field of neural engineering.

To further evaluate women’s representation in the field of neural engineering, we applied the Balanced Citer tool (Dworkin et al., 2020) to papers published in the Journal of Neural Engineering (JNE) and IEEE Transactions on Neural Systems and Rehabilitation Engineering (TNSRE) for the years 2020–2022. The Balanced Citer tool is an open-source tool that probabilistically infers the gender of the first and last authors of papers based on the author names. Results from the tool suggest that in JNE 29.3% of the 799 papers that were classifiable (out of 1,073 total papers) likely have female first authors and 16.4% likely have female last authors. In IEEE TNSRE, of the 572 papers out of 897 that were classifiable, the tool classified first authors as female in 26.4% of the papers and last authors as female in 19.3% of the papers. Across both journals, 59.8% of papers published since 2020 had first and last authors who were both male, while only 5.5% of papers were written by first and last authors who were both female. It is reasonable to expect that most first authors are likely trainees while last authors are likely faculty-level investigators. Authorship statistics seem to reflect gender disparities across career stages, at least in this limited sample of data. Broader analysis of authorship demographics would be informative for our field, but is beyond the scope of this work.

### 3.2. Identity-related stereotypes, harassment, and bias

Children are exposed to gender stereotypes at young ages, and these stereotypes can influence career aspirations (Makarova et al., 2019). Math and physics, for example, have been associated as masculine subjects by both male and female secondary school and college students and teachers (Nosek et al., 2002; Makarova and Herzog, 2015). This presents a major barrier for female students; seeing their identities as at odds with STEM subjects can reduce their confidence in their ability to perform well in these subjects, and deter them from pursuing STEM altogether. Moreover, these stereotypes can be reinforced by negative performance that is itself a result of the stereotypes. This phenomenon, known as stereotype threat, occurs when awareness of negative stereotypes about one’s performance in a situation adds a cognitive burden that negatively affects actual performance (Spencer et al., 2016). These stereotypes can also contribute to what has been termed “impostor phenomenon,” or the perception that one does not belong in their role or deserve the success they attain. Dr. Orsborn described the cognitive load involved in second-guessing whether barriers are due to her actual skillset, being a woman, or some other factor such as personality. She recalled encountering skepticism of her expertise in different areas depending on the background of the people she was interacting with. As neural engineering relies on knowledge across a broad range of disciplines, navigating these doubts can be particularly difficult. Several of our interviewees noted the emotional toll associated with defending their identities and proving that they belong in their roles. This experience can have lasting ramifications beyond the immediate emotional toll, affecting individuals’ productivity and ability to perform in their roles.

Harassment based on gender, sexual orientation, and/or race also takes a large emotional toll and can hinder career progress for women at these intersections. This harassment can take the form of overt sexist or racist abuse, but often also takes the form of microaggressions: subtle, everyday interactions that portray a demeaning attitude toward a marginalized identity. One interviewee experienced “microaggressions, just being a woman and also identifying as gay, from my peers.” Furthermore, the interviewee reported that the time diverted from research toward defending her identity and competence resulted in her generating fewer scientific publications. Microaggressions can often go unnoticed by people outside of the targeted marginalized identity, and may not even be perceived as demeaning by their perpetrators. However, their effects on marginalized graduate women can seriously and negatively impact academic progress, with decreased productivity and increased thoughts of dropping out, as well as detrimental consequences to mental health, with increased depression and anxiety from having their identities as scientists constantly challenged (Miles et al., 2020; Wilkins-Yel et al., 2022).

Women and gender minorities who are LGBTQ (lesbian, gay, bisexual, transgender, and queer) may experience additional discomfort in STEM fields (Hughes, 2018). The gendered expectations common in STEM fields can make it particularly difficult to be gender non-conforming in the field due to gender stereotypes as well as discrimination such as misgendering (Goldberg et al., 2021). Moreover, people who have changed their names to reflect their gender identity may be in danger of their non-cis identity being revealed against their wishes due to their publication records retaining a previous name, a potential additional stressor for trans people in academia (Gaskins and McClain, 2021). Joni Wallis, who is a professor at the University of California, Berkeley in the Department of Psychology and the Helen Wills Neuroscience Institute and is a trans woman, highlighted some additional issues relevant to trans students in an interview published in Neuron (Wallis, 2021). She pointed out that food insecurity, homelessness, and mental health concerns affect trans students more often than cisgender students and are likely to affect academic performance. Furthermore, gender-affirming care is rarely supported on graduate student healthcare plans. Gender transition can also be a time-consuming process that may disrupt a more traditional academic career timeline.

In addition, women of color are subject to multiple sources of bias, experiencing both race- and gender-based barriers (Ireland et al., 2018) in a way that exceeds the combined individual impact of race-based and gender-based discrimination (Crenshaw, 1989; Carbado et al., 2013). A 2018 report showed that across all engineering faculty in the United States, only 28 full professors were Latina women, and only a subset of those were in biomedical engineering (Arellano et al., 2018). Furthermore, as of 2021, there were 28 American Indian full professors (genders unspecified) across all engineering faculty. Only 4 American Indian female students received doctorate degrees in engineering in 2021, a mere 0.03% of engineering doctorates received that year (American Society for Engineering Education [ASEE], 2021a). At predominantly white institutions, Black women are often stereotyped as loud, angry, unserious, and unintelligent by their white classmates, leading to exclusion from social groups and classroom participation (Neal-Jackson, 2020). Many Black women in academia have described a lack of professional development and emotional support from their advisors (Spencer et al., 2022). A previously published collection of interviews of Black women who are graduate students revealed that many felt isolated during their engineering or computing graduate school experience as they were often the only, or even the first, Black woman in the program (Telfer, 2022). A sense of community can have a large impact on their success in STEM fields; a disproportionately high number of the Black women who obtain bachelor’s degrees in STEM fields graduate from Historically Black Colleges and Universities (Perna et al., 2009). For Ms. Jones and several of her Black colleagues in neuroscience and neural engineering, this was the impetus for their initiative, Black in Neuro. In our interviews, Ms. Jones described forming Black in Neuro in response to some of these feelings of isolation. She and her co-founders developed a network of Black students and post-doctorates in the field, with the support of a few faculty members. Black in Neuro members and attendees of the workshops delivered by the organization praised the sense of community that this organization fostered.

For disabled women in the field, their experiences of alienation are exacerbated by the fact that the experiences and needs of disabled researchers are often overlooked throughout STEM environments. Unfortunately, research labs in neural engineering and other STEM fields are lacking in basic accessibility measures that would enable disabled researchers to fully participate (Thurston et al., 2017). Dr. Orsborn stressed that “a lot of the ways we do current lab science and experiments is incredibly not inclusive to people with physical disabilities.” This is particularly troubling in a field like neural engineering, wherein much research is motivated by the effort to improve the lives of people with neurological diseases and/or injuries. Given this context, the lack of accessibility in neural engineering at best belies these stated motivations and at worst can make the field look hypocritical. Some small adjustments to improve the accessibility of lab spaces, such as assistive technology to facilitate using lab tools or adjustable table heights, can make a lab environment more inclusive to people with physical disabilities (Jeannis et al., 2018).

Yet another form of identity-based marginalization with underappreciated effects is immigration status. Dr. Castagnola expressed that international trainees often feel trepidation about reporting conflicts or problematic behavior with or about their advisors because the status of many US visas depends entirely on maintaining employment; losing one’s job could result in deportation. When this power imbalance intersects with gender, it can mean that faculty members who are unsupportive of or even outright toxic toward female students and post-doctorates continue without appropriate consequence, thereby perpetuating inequities. This is most striking in the case of sexual harassment and other forms of abuse. Dr. Castagnola noted that in surveys she had conducted about people’s stories of inequalities in STEM, international women trainees reported a reluctance to report sexual harassment due to the dependence of their visas on their advisors. The power imbalance experienced by international trainees can also make it difficult for these trainees to report other forms of abuse and discrimination; balancing these pressures can take a toll on a person’s ability to perform in their career (Lee and Rice, 2007).

### 3.3. Mentoring, networking, and authorship

Mentorship and networking are two central elements of career development, and are often positioned as key interventions to improve career trajectories for women in STEM (National Science Foundation [NSF], 2020a). Nevertheless, structural barriers stand in the way of women receiving adequate mentorship and networking opportunities, contributing to the attrition of women from academia. Women are offered less career mentorship by science faculty (independent of faculty gender) than men (Moss-Racusin et al., 2012). As Dr. Ting said, “not having mentors means that people leave.” Dr. Vazquez emphasized the need for institutional mentoring networks to support early career faculty members and students. Early career faculty are in particular need of formal guidance, with one study finding that 25% of new principal investigators (PIs) felt they had no mentorship, with more negative consequences on women (Acton et al., 2019). Moreover, for LGBTQ people and gender minorities, the lack of LGBTQ role models in engineering fields can contribute to a hostile work environment, with entrenched views of STEM fields as heteronormative and masculine. This leads many LGBTQ engineering students and faculty to keep their identity closeted to fellow colleagues (Bakka et al., 2021). Mentors of women and gender minorities do not necessarily need to share their gender identity, but “perceived similarity,” or an alignment of outlooks, perspectives, and values between mentors and mentees is important to the quality of the mentoring relationship (Eby et al., 2013; Hernandez et al., 2017). Mentors of women and gender minorities must be willing to advocate on behalf of their mentees, especially in situations where identity is a factor. Although supportive mentors can be any gender, women at more advanced career stages can provide unique perspectives and career support to women trainees. Difficulty in finding this support led several of our interviewees to seek out opportunities to work with other women. Drs. Orsborn and Vazquez both noted that most of their mentors have been men and Dr. Ting recounted that a lack of mentorship contributed to her feeling unsupported early in her career. This led her to seek out mentorship from a woman supervisor, which was instrumental in rebuilding her self-confidence. Dr. Orsborn identifies mentorship and networking as an explicit goal of the WINE forum, noting the power of such an initiative to illuminate some of what is sometimes described as the “hidden curriculum:” unwritten expectations for behavior that people do not receive instruction on during formal training. The “hidden curriculum” can include informal knowledge about areas such as networking, job interviewing, negotiation, and navigating other career hurdles.

This “hidden curriculum” is particularly challenging to navigate for students who are first-generation college students (students who do not have parents that attended college). This group of students is also more likely to include students with minoritized racial and ethnic backgrounds than non-first-generation students (McCarron and Inkelas, 2006). First-generation students lack access to institutional knowledge in higher education and consequently have lower grades and higher dropout rates, an effect that is strongest in STEM fields (Canning et al., 2020). Furthermore, impostor phenomenon is exacerbated for first-generation students in classroom environments that are very competitive, a hallmark of many STEM subjects (Canning et al., 2020). Ms. Jones said that as a first-generation student, “I had no idea what I was getting myself into,” but mentorship and resources from other women were very important to gaining clarity about the field and about career opportunities. As a former first-generation student herself, Dr. Vazquez is committed to mentoring other first-generation students now, recognizing that everyone in academia must work hard but for people who already know how navigate academia, “it’s like being on the highway vs. a four-wheeler in the mountains.” This understanding shaped her mentorship style, which focuses less on traditional barriers of entry, such as GPA, and more on willingness to learn, opening up her lab to students who may have otherwise been denied opportunities.

Although mentorship is essential for women at the beginning of their careers to navigate the “hidden curriculum,” women’s careers can be disproportionately affected by mentoring responsibilities with limited public or professional recognition (Howard-Vital and Brunson, 2006). For example, as one of few women in neural engineering at the full professor level, Dr. Ting writes many tenure letters for junior women faculty and provides advice on negotiations. In addition to typical mentoring responsibilities, she feels a responsibility “to help the women…deal with salary equity and retention offers,” and to share other similarly hidden knowledge. This work is essential to help early career women navigate the field, but places an additional service burden on women at later career stages. Facilitating peer mentoring and finding role models for women at earlier stages of their careers can help build community and provide support while lessening this service burden.

Professional networks also have direct impact on access to opportunities for career advancement, such as invitations to give talks, which can bolster a CV and provide greater visibility for a researcher’s work. Such invitations are often extended through formal or informal networking, which disproportionately leads to the exclusion of women (Nittrouer et al., 2018). Dr. Vazquez pointed out that opportunities such as invited talks are only available to historically marginalized researchers if senior academics specifically make an effort to advocate for a diverse group of junior academics, placing “the onus is…on full professors in neural engineering to look for people who are not yet full professors…to make them visible, and that makes all the difference.”

How women and gender minorities are perceived by their superiors, peers, and students all play a role in career success. Ms. Jones shared that “one of the biggest frustrations I’ve ever dealt with as a woman…is not [being] seen as credible or taken seriously by my contemporaries.” Interpersonal interactions with students reveal further biases against the perceived abilities of women. Student evaluations of teaching are important for tenure and promotion. Women instructors are more likely to receive sexist and abusive comments than men (Heffernan, 2022). Students are also more likely to address their woman professors less formally. Dr. Vazquez described being called “Ms. Vazquez” by the same students who addressed her male colleagues as “Dr.” However, when recounting these incidents to her male colleagues, they did not see a problem with the students’ error. Mis-titling has been documented in the literature. For example, men introducers are substantially more likely to introduce men using their formal title, whereas women introducers are equally likely to introduce both men and women using formal titles (Files et al., 2017). Mis-titling women further reduces the perception of competence, can undermine women’s authority and accomplishments, and takes a further emotional toll on women to combat these biases.

Women are also more likely than men to have authorship disputes, both regarding being listed as an author and the order of authors (Ni et al., 2021). This indicates that women are receiving less credit than they deserve for the work that they complete. Conversely, men self-reported that they often receive more credit they deserved (Ni et al., 2021). One of our interviewees recounted that she developed and completed a project for her master’s thesis, but her work was used as the basis for a publication led by two white male students, on which she was not included. Because neural engineering is a highly collaborative field, projects may involve large teams, and it is particularly important to be aware of women’s contributions. These projects also often include more men than women. For example, the Brain Research Through Advancing Innovative Neurotechnologies (BRAIN) Initiative reported that 47% of its grants were awarded to multi-PI teams (Crawford et al., 2022). The largest area of research expertise by applicants was engineering. More than half the teams were male-only, while 34.7% of teams were mixed male and female. Only 1.3% of teams were female-only. This report highlights two things: the need for more female PIs on research teams, and that within these highly collaborative teams, researchers need to reassess how they assign credit. Publications are a marker of academic success; by not receiving appropriate credit in publications, women’s careers are being directly and negatively impacted by these disputes. Mentors of women need to be aware of these biases and conflicts disproportionately faced by women and take action to correct them. Dr. Ross expressed gratitude to the “incredible advocates [she had] along the way who [had] spoken up when they saw [she] was being treated differently as a woman in STEM and who [had] ensured that [she] had opportunities to continue to grow as a leader in this field.”

### 3.4. Resources and salary

The aforementioned lack of mentorship and the obscurity of the “hidden curriculum” have material consequences as women advance in their careers. For example, these issues can directly impact women’s access to the financial resources needed to support their careers. In salary and lab start-up package negotiations, women who lack mentors to help guide them through the process may find themselves ill-equipped to negotiate their offers. Dr. Orsborn pointed out that “people don’t necessarily talk super concretely [about] what you can and can’t ask for, [or] how those negotiations work.” Similarly, Dr. Ting observed that women often get less money and resources because they do not know how to ask for it in the “right way.” However, even when women are equipped to negotiate, they are less likely than men to be well-received (Bowles et al., 2007).

The result of this bias in resource allocation is that women with doctoral degrees have lower salaries in full-time employment (National Science Foundation [NSF], 2020b) and at all levels of academia (National Science Foundation [NSF], 2017b). Dr. Ting noted that pay equity remains a particularly significant concern for historically marginalized individuals who are outside the “inner circle” of academia (or who lack strong, influential professional networks). For her, having a female Department Chair has helped to ameliorate pay equity issues, highlighting the importance of representation at all levels of academia. Dr. Orsborn noted that lower salaries have a particularly profound effect on those who come from a lower socioeconomic background. In those cases, lack of pay equity and low salaries can have a major impact on their decisions about advanced trainee and career opportunities. In fact, Ms. Jones identified salary and finances as factors that contributed to her decision to leave academia.

### 3.5. Familial and service obligations

Women are often subjected to additional responsibilities and expectations that can hinder the advancement of their research careers. These can take the form of personal responsibilities (such as child and family care) that are insufficiently supported by institutions, as well as expectations that women will take on non-research responsibilities within their professional roles. Women are often penalized in their careers for parental and familial responsibilities. Beginning with pregnancy and childbirth, women who have children face insufficient maternity leave policies. For instance, one interviewee reported having no institutional maternity leave available during her first pregnancy. She was fortunate enough to have a Department Chair who accommodated her needs, but a lack of institutional support leaves academics dependent on the goodwill and understanding of individual Chairs and Department Heads as they prepare for parenthood. Furthermore, lack of support at an early stage of their careers may leave women playing catch-up when applying for later positions and promotions.

For academics with family care responsibilities, a lack of affordable care options can significantly hinder their ability to advance in their careers. Dr. Orsborn suggested that to improve equity and conditions for women, institutions should pay for childcare or at least guarantee onsite child and elder care availability for all employees. Fortunately, in recent years institutions have worked on improving resources and opportunities to support families. Many institutions now offer onsite child and elder care, tenure clock extension, and pause teaching duties for a semester to a year (Carr et al., 2019). However, these resources are not always accessible. Onsite child and elder care are often filled to capacity, and prices can be untenable, especially for students and post-doctorates (Monroe et al., 2014). Moreover, they are often underutilized due to an expectation of negative personal or professional repercussions (Carr et al., 2019). In fact, an interviewee reported facing negative professional consequences for her maternity time when she returned, having to increase her teaching load to make up for the time she had been away, and being removed from another professional advancement opportunity. Interestingly, she recounted facing the most criticism from older women faculty who had to endure similar treatment when they themselves became parents; their attitude seemed to be that enduring those conditions was a rite of passage in this career path.

In addition to balancing personal responsibilities, women are often expected to take on greater responsibilities in other dimensions of academia, most notably in their teaching load and service work (Misra et al., 2021). Because neural engineering is a highly interdisciplinary field, many neural engineers have multiple departmental affiliations, which can multiply the number of service requests. New women PIs are expected to spend more hours teaching and participate in more committees as compared to their male colleagues (Acton et al., 2019). Despite the additional time commitment required of female academics, they receive poorer responses on teaching evaluations than their male colleagues, regardless of actual performance (Heffernan, 2022). This bias is further exacerbated for professors who are outside of the following demographics: white, native English speakers, able-bodied, perceived as heterosexual, and between the ages of 35 and 50 (Heffernan, 2022). Because teaching is frequently required for tenure, biased student evaluations may have a substantial impact on women’s careers. Dr. Vazquez recalled how societal gender roles of women impacted her teaching evaluations. Students expected her and other female faculty “to be like kindergarten teachers.” In other words, students expected her to selflessly prioritize helping them and to provide extensive additional resources, even outside of class and designated office hours. Dr. Vazquez’s male colleagues attached a similar stereotype to her, believing that her position as a young female faculty member made her more relatable, even to young male students. These stereotypes place an undue burden on female faculty members to spend more time and energy on coming across as kind, approachable, and generous in order to be perceived as good teachers.

Women are assigned more non-promotable tasks than men, that is, tasks that are necessary to keep an organization functional, but may be viewed as secretarial rather than research work (Lester, 2008; Alemán, 2017). Dr. Ross recalls “many experiences where [she] was the only woman in the room and was therefore asked to do things like take notes or to organize an event.” Holmes et al. found that women spent less time doing experimental tasks and more on note-taking when in mixed-gender groups (Holmes et al., 2022). In a research setting, these types of assignments may include day-to-day tasks that maintain a lab, such as ordering supplies, scheduling lab meetings, or animal upkeep. Research productivity and the lab’s physical and intellectual environment can depend on the completion of these tasks. A series of interviews with biology Ph.D. students found that women were more likely to engage in service and lab management activities than men (and among men, racial and ethnic minorities contributed more to service than white men). The women interviewees described being overburdened by lab organization and undergrad mentorship responsibilities, roles that carry no additional financial compensation (Miller and Roksa, 2020). This presents a twofold problem, and therefore both factors must be addressed: firstly, labs and institutions must recognize such service work as crucial and compensate for this work accordingly, and secondly, these roles must be intentionally and equitably distributed to reduce the disproportionate burden on women and people of color.

As institutions and organizations increasingly begin to recognize the need for initiatives to promote diversity, equity, and inclusion (DEI), the burden of these tasks disproportionately falls on women and gender minorities. Across many fields and industries, the onus for promoting, organizing, and participating in DEI efforts falls largely on the historically marginalized groups who are most affected. These efforts are usually extra responsibilities taken on outside of their formal job roles, which can impact their productivity as researchers (Casad et al., 2021). Additionally, women and minorities are frequently penalized for promoting diversity initiatives (Eliezer and Major, 2012; Patton and Bondi, 2015). In order for these efforts to be sustained equitably, members of the majority must also make these initiatives a priority. Ms. Jones explains that in her initiative, Black in Neuro, “the organizers are Ph.D. students themselves,” who need the support of their advisors and their departments to promote and financially support their work. The survival of these services relies on active participation, continued promotion and support by not just the organizers, but their community as a whole. Dr. Vazquez notes that “women do a disproportionate amount of service,” and that this has not improved in the last decade or two. As women and minorities typically start with a higher service burden, in addition to the mentorship burden, this load can be reduced if white men take on additional service responsibilities. The disproportionate number of women participating in diversity and service work has been reported previously (Casad et al., 2021; Misra et al., 2021), and is also apparent in the authorship of diversity-related publications. In our reference list, 66 out of 92 references were successfully categorized by predicted gender. Of the categorized references cited in our paper, 69.7% had first authors who were women, and 66.7% had last authors that were women.

## 4. Contextualization and expansion of interview themes

In this section, we expand on themes that the interviewees raised, but discussed only within the context of their own experiences. Further discussion into these topics is crucial to understanding their breadth and impact. During the interviews, all the women emphasized that although neural engineering is an imperfect field, they have also found support networks, in part facilitated by the greater proportion of women compared to other engineering disciplines. Dr. Orsborn expressed that “it’s not an accident that [she] shifted toward a field with more representation,” and Dr. Ting “tried it all and found there were no perfect fields.” It was clear from our interviews that women in neural engineering must overcome barriers both professionally and personally in order to succeed in academia. Even once success is achieved, women put substantial effort into service work in addition to their academic labor. However, as Dr. Castagnola mentioned, it is common that talks, panels, and committees that aim to address these issues have few male attendees, and those who do attend are often part of underrepresented groups themselves. Taking action to reach equity must be a priority for the majority. Beyond the issues brought up by our interviewees, our literature review revealed additional, broader barriers to women in engineering. Studies show that women in STEM fields are perceived as less competent, are less likely to be hired than men with the same qualifications, and advance to higher positions more slowly than men (Moss-Racusin et al., 2012; Wijnen et al., 2021). Despite these facts, there is a misconception that the presence of more women in biological fields compared to fields such as mathematics means that biomedical and neural engineering do not exhibit the same sexism that is present in other STEM fields (Llorens et al., 2021). Unfortunately, as our interviews showed, this field is not an exception. At the current rate of change, the field of biomedical engineering will first reach an equal gender balance at the professorial level in the year 2067, which is an unacceptably delayed timeframe for gender parity (Figure 3C). Furthermore, these barriers are exacerbated for women and gender minorities who also belong to other underrepresented groups. Failure to include women and gender minorities as researchers is not only inequitable, but will also be detrimental to research outcomes. Women and other historically excluded groups make more novel scientific contributions, but their work is less recognized; we may be losing valuable discoveries and solutions when we push away women researchers (Hofstra et al., 2020). To improve equity in neural engineering, several areas must be addressed.

### 4.1. Sense of belonging

A repeating theme that arose in many of our interviews and in the literature is the importance of a sense of belonging in academia and in the field of neural engineering. Many of our interviewees described building their own support networks, either by seeking out female mentors or by building peer mentorship networks, such as the WINE forum or Black in Neuro. Evidence in the literature suggests that the presence of female mentors (including peer mentors) contributes to greater retention and positive outcomes for science and engineering undergraduate students (Dennehy and Dasgupta, 2017; Moghe et al., 2021). Among women undergraduate students who do achieve a sense of belonging in their engineering program, it is often thanks to the support of their peers through organizations like SWE (Society of Women Engineers), NSBE (National Society of Black Engineers), and ASME (American Society of Mechanical Engineers) (Wilson and VanAntwerp, 2021). However, in the post-graduate years where these close-knit organizations are not as available, women engineers in graduate school and the workplace report increased feelings of isolation (Wilson and VanAntwerp, 2021). This decreased sense of belonging trends with the declining proportion of women in the higher stages of their academic careers, especially for women of color.

The consequences of inadequate support for women of color are evident in the low representation of historically marginalized groups. Universities can address this shortage by implementing policies and programs that involve students in academic programs, support faculty mentorship relationships, and avoid grouping all minorities under a single umbrella. Efforts that create community around shared identity can be particularly impactful, especially if they account for the ways that identity and culture inform individuals’ engagement with academia. One study focusing on Latina engineers found that those who placed higher value on self-sacrifice, based on cultural or familial expectations, were less likely to pursue STEM careers, as were those who perceived their university classroom environment as hostile (Castellanos, 2018). This is one example of a way in which cultural background can inform how individuals interact with the prevailing academic environment and make career decisions. Institutions can mitigate some of these barriers by intentionally supporting diverse forms of mentorship and offering support for a diverse array of cultural values, backgrounds, and strengths.

Unfortunately, these mentorship and social networks are difficult to build; women and minority students’ requests for mentorship are more likely to be ignored than those of their white male counterparts (Milkman et al., 2015), and women are offered less career mentorship by science faculty than men (Moss-Racusin et al., 2012). Meanwhile, a lack of mentorship can have a pronounced impact on women’s experience of their work, with women who lack mentors expressing less optimism about their future than women with mentors and men both with and without mentors (Acton et al., 2019). Furthermore, mentorship networks can be critical to reveal the “hidden curriculum” and allow women to reach their career goals.

Important as they are, these support networks can fall short. Alarmingly, women’s professional and mentoring networks, where they do exist, have fewer high-status connections, which may result in poorer future job prospects (Blommaert et al., 2020). In an analysis of scientific collaborations in engineering fields, it was found that women occupy less central positions in their scientific networks (Ghiasi et al., 2015). Although engineering is a male-dominated field, and therefore it is expected that engineers will collaborate with men more often than women, it was found that 38% of female engineers and 50% of male engineers have no female co-authors (Ghiasi et al., 2015). This is unsurprising, given that the BRAIN Initiative reported more than half of the research teams were all-male, and almost no teams were all-female (Crawford et al., 2022). However, mixed-gender collaboration teams have higher productivity and are more central to the scientific network (Ghiasi et al., 2015), suggesting that it would be beneficial to all co-authors to seek out these collaborations. Neural engineers in particular may benefit from diverse support networks due to the multidisciplinary nature of the field, which requires collaboration across other areas of STEM that may each have their own norms and biases.

### 4.2. Biased perceptions of women’s work

Bias in how women scientists are perceived is pervasive and is exhibited both during day-to-day evaluations of women’s work as well as the hiring process. However, this systemic bias is difficult to evaluate on an individual level; as several of our interviewees pointed out, attempting to assess whether a particular experience is due to sexism or other factors can create an additional mental burden for female scientists.

One area where bias against women researchers is pervasive is publications. Discrepancies in publishing begin early in an academic’s career, as male Ph.D. students in engineering and the biological sciences publish more papers than female Ph.D. students in the same fields (Lubienski et al., 2018). Women are also underrepresented in high-profile journals (Shen et al., 2018) as well as the more prestigious first and last authorship positions (Ni et al., 2021). Furthermore, although co-first-authorship purports to equally share credit between several authors, when women in biological sciences are co-first-authors with men, they are disproportionately likely to be listed second (Broderick and Casadevall, 2019). When published, women are less cited, particularly by men (Dworkin et al., 2020), and Black women are the most under-cited (Bertolero et al., 2020). Unfortunately, the under-citation of women authors is not decreasing over time (Dworkin et al., 2020). These disparities in citations then result in, and are reinforced by, a gender disparity in prestigious awards (Melnikoff and Valian, 2019).

Bias also affects the amount of early career funding a researcher receives; women receive fewer financial resources to support their scientific careers. This neglect begins early; female undergraduate and master’s students who apply for scholarships are less likely to be funded compared to their male counterparts (Wijnen et al., 2021). When gender-related information was removed, applications by men and women were evaluated to be equal in quality, revealing a persistent bias against funding female students. Even more alarming was that the gap between the number of applications funded for men vs. women widened over time, demonstrating that the bias against female students was becoming more pronounced (Wijnen et al., 2021). Acquiring one grant increases the chances of acquiring future funding (Reinhart, 2009). Therefore, hindering women’s ability to obtain scholarships and grants at earlier stages in their careers sets them back for future successes compared to their male counterparts. Gender inequities due to reviewer biases also result in women receiving fewer and smaller grants (Steinþórsdóttir et al., 2020). A study investigating National Institutes of Health (NIH) funding of first-time PIs showed that women received a median of $39,106 less in grant funding than men across all institutions and grant types (Oliveira et al., 2019). Funding discrepancies arise in part because grant reviewers rate male PIs more positively than female PIs, even though the research components of grants written by male and female PIs are not scored differently (Witteman et al., 2019).

Funding discrepancies can have a pronounced impact on early-career faculty as well. Indeed, female junior faculty experience resource discrepancies in start-up funds for their lab (Acton et al., 2019). Data from 2012 to 2014 indicate that female biomedical researchers receive less than half the start-up support from their institutions compared to male biomedical researchers, regardless of degree and years of experience (Sege et al., 2015). Less institutional support may result in lower publication rates (Duch et al., 2012), further impeding women’s career success. Furthermore, female junior faculty are often given smaller lab spaces (Else, 2019). While some changes have been made to correct the inequality in start-up funds and lab space, a recent study suggests that these disparities are still prevalent (Acton et al., 2019). This may be in part due to women being less likely to negotiate (Fischer and Bajaj, 2017). While this may seem to suggest that women should simply negotiate more aggressively, evidence shows that hiring committees penalize women who negotiate (Bowles et al., 2007).

In job applications, women must be higher-quality applicants than men to be viewed as equally qualified (Wijnen et al., 2021). Unfortunately, all of the above factors conspire to make it more difficult for women to build equally impressive resumes to their male colleagues and further undermine the potential of women to be hired. Indeed, in studies of candidates for STEM academic jobs with identical resumes with either typically male or female names, male candidates were typically viewed as more competent and more hirable (Moss-Racusin et al., 2012; Eaton et al., 2020). This effect is stronger when combined with race, as Black and Latina female candidates have a significant disadvantage compared to all other groups (Eaton et al., 2020). Job applications may come with additional challenges for trans and non-binary scientists, who may remain closeted during the job application process out of concerns it will affect their career progression (Goldberg et al., 2021).

### 4.3. Societal factors

In addition to the aforementioned factors, there are certain societal and external factors indirectly related to research that hinder women’s academic career advancement. In recent years, the gender gap in authorship has been compounded by the COVID-19 pandemic, which has resulted in a decrease in paper submissions by women compared to men and had a particularly negative impact on women of color and women early in their careers (Blackburn, 2022). Furthermore, women submitted significantly fewer grants to the NIH compared to men in 2020 (Roubinov et al., 2022). Women are often expected to take on the bulk of family care responsibilities; the COVID-19 pandemic and lockdowns forced many women to care for their families during the day, sacrificing their working hours and productivity.

Sexual harassment, which disproportionately affects women, is rampant in STEM fields, and more than half of women faculty and staff in academia report experiencing it (Committee on the Impacts of Sexual Harassment in Academia et al., 2018). This phenomenon is even more severe for women of multiple historically marginalized identities. Despite its prevalence, formal action is only taken 2–25% of the time, for fear of retaliation by the perpetrator and expectation of a bad investigative result (Committee on the Impacts of Sexual Harassment in Academia et al., 2018). As Dr. Castagnola mentioned, it is even more difficult for non-citizen trainees to speak out as retaliation could mean deportation. The severity of this harassment is directly correlated with greater stress, anxiety, and dissatisfaction with one’s job, leading many to exit their positions. For those who choose to stay, many cite their women colleagues as their primary support structure, not their supervisors or department leaders (Committee on the Impacts of Sexual Harassment in Academia et al., 2018). In order to retain women in engineering, especially in higher career stages, it is imperative that institutions adopt a zero-tolerance policy on sexual harassment and that crude jokes and sexist comments are taken seriously. Additionally, it is also the responsibility of bystanders witnessing this behavior to hold perpetrators accountable; the burden of correcting or reporting inappropriate behavior cannot once again be placed solely on the victims.

We selected interviewees who were still in the field of neural engineering and who had formed either a formal or informal initiative to help other women in the field. Therefore, our pool of interviewees is subject to a survivorship bias, as they were inherently a group of women who have found it possible to overcome existing barriers themselves. It is worth noting again that since the time of the interviews, Jones (2022) has left academia, and attributed her decision to a combination of the issues we have discussed above. Additionally, this is a group of women who have both noticed that these barriers exist and who would be inclined to feel that they are surmountable with additional support. This was evident in some interviews. Before the fruits of these initiatives can be harvested, however, Dr. Castagnola suggested that unfortunately in the meantime, young female students may have to build emotional strength and endurance to overcome said barriers.

## 5. Limitations

This paper focuses on the experiences of women at academic institutions in the United States; however, we provide evidence from literature that describes similar experiences by women globally. Although many of these problems are endemic to neural engineering as a whole, the specific systemic barriers and the most effective interventions will vary in different countries.

Here, we broadly acknowledge intersectionality through the context of our interviewees’ experiences and discuss issues pertaining to race, queerness, immigration status, and more. However, each of these intersections as well as other issues related to intersectionality could constitute entire papers in their own right, and are not comprehensively addressed here.

Although both our interviewee list and author list include queer women, all women on both lists are cisgender. Furthermore, the vast majority of literature compares the experiences of cisgender men and women, and very rarely explores the experiences of trans and genderqueer scientists. Studies on queer scientists are scarce, likely in part due to the relative recency of legal same-sex marriage in the United States and the rapid changes in cultural acceptance of queerness. This rapidly changing legal landscape has a significant effect on career choices of queer scientists, and particularly trans and gender non-conforming scientists, who may have to evaluate state-level politics as a key component of the positions that they consider. In many demographic datasets, including those we used from ASEE and the NSF, the data are limited to binary genders or sex (woman/man or female/male). These demographic data form the basis for many further studies and failing to include LGBTQ identities creates a gap in any study that refers to the data. In fact, the NSF and the NIH both fail to mention LGBTQ people as an important part of a diverse workforce, despite explicitly calling for the inclusion of many other underrepresented groups (Freeman, 2018; National Institutes Of Health [NIH], 2019; National Science Foundation [NSF], 2020a). In order to effectively confront the unique forms of bias faced by trans, non-binary, and queer scientists, it is important to collect data about their experiences, needs, and whether they remain in the academic STEM workforce. Future studies should avoid this false gender binary, and more study is needed regarding professional and personal factors in career development for queer scientists (Freeman, 2018).

The data we collected from annual and biannual reports from ASEE and the NSF showing the proportion of women at each academic stage contained gaps in reporting, especially over time. Digital reports from ASEE were not published prior to 2005. For data prior to 2005, we relied on reports published by the NSF, which contained gaps in reporting the demographics for bachelor’s degree recipients for mechanical and electrical engineering, and no faculty demographics for sub-fields in engineering. Additionally, since biomedical engineering is a relatively new field, we were unable to find demographic data prior to 2005 for bachelors and doctorate degree recipients (except for one time point in 1995), and prior to 2001 for faculty. For biological sciences, demographic information for recipients of bachelors and doctorate degrees was intermittent between 1979 and 2000, after which reporting was consistent. Demographic information of post-doctoral fellows was available for each year between 1979 and 2006, after which reports became biannual. Faculty demographics were available starting in 2003 but were subsequently reported every 2–3 years. It is also worth noting that we chose to report data from biological sciences because data for sub-categories such as neuroscience were sparse and inconsistently reported. In the future, this demographic information should be more consistently tracked and reported, and databases publicly available, for more thorough analyses to be performed.

## 6. Recommendations

It is evident that there are still pervasive barriers to women’s advancement across career stages in neural engineering. Based on current trends, biomedical engineering, and likely neural engineering, will not reach gender parity until 2067. This points to a problem that is too big to “wait out,” and that everyone must take responsibility for. Below, we have compiled a list of recommendations to address current barriers and promote women’s advancement in the field. These recommendations focus on issues that were identified in our interviews and literature, and can be implemented at the individual, institutional, and societal levels. Because many of the challenges experienced by women and gender minorities in neural engineering are also common in other STEM fields, many of these recommendations are broadly applicable. Nevertheless, we believe it is important for the field of neural engineering specifically to adopt and deeply engage with these recommendations. In order to properly engage with and implement these recommendations, readers will have to work with their institutions and departments to identify the best approaches for their situation. As a relatively new field, neural engineering can proactively adopt these recommendations, thereby developing a structure wherein these principles are inherent and central to a scientific field. In doing so, this field could serve as a model for other STEM fields. Additional resources and organizations dedicated to the support of women in neural engineering are provided in Supplementary Tables 1, 2.

### 6.1. Actively engage in continued learning

In advocating for gender equity, it is imperative that men, who are more likely to hold positions of power and who face fewer consequences than women do for challenging the *status quo* (Eliezer and Major, 2012; Patton and Bondi, 2015), take an active role. As one example, panels, research, and conferences focusing on diversity are often disproportionately organized and attended by women and people from historically marginalized identities. A first step for people who are not directly affected by these issues (in this case, men, and especially white men) is to actively seek continuing education about the challenges that affect their marginalized colleagues. Equity and inclusion are integral to neural engineering as it is crucial to develop technologies and therapies that meet the needs of everyone, regardless of gender, race, age, ability status, socioeconomic class, etc. (Goering et al., 2021). It is therefore important to organize at the lab, departmental, and institutional level to implement ways for everyone to engage in advocacy. Examples of successful approaches in existing neural engineering labs include requiring all lab members to develop and implement plans for engaging with DEI work and developing a regular trainee-driven speaker series and discussion group to expose lab members to relevant issues and information. Because of shifting social perception and changes in the necessary resources and support, the path to equity is dynamic. Therefore, effective advocacy requires consistent engagement and sustained effort.

### 6.2. Increase access to mentorship

Mentorship is critical to the retention and success of female trainees, and promotes a sense of belonging in the field. Diverse mentorship and support networks are critical to fully support women’s multifaceted personal identities and to span the multiple academic fields that coalesce to create the discipline of neural engineering. Senior neural engineers should seek out opportunities to mentor female trainees. Male faculty members must take the initiative to be well-informed on the barriers that may specifically impact their marginalized mentees in order to provide effective mentorship. At the institutional or lab level, facilitating peer mentoring and networking opportunities for women at early career stages can help build community and provide support without increasing the service burden on the few women in senior positions. Institutions must financially and materially support initiatives designed to provide mentorship and networking opportunities to women to ensure that such initiatives last and succeed.

### 6.3. Advocate for and amplify the work of female researchers

Success in STEM fields remains heavily contingent on tight professional networks that enable access to opportunities, which often keep women on the periphery. In group discussions, ensure women are not interrupted and give credit to their contributions. Senior faculty members can also amplify women by recommending junior female researchers for speaking opportunities and awards and encouraging women to participate in leadership and career development programs. Institutions can help by providing and supporting such programs.

### 6.4. Explore alternative evaluation practices to improve diversity

Structural biases often go unnoticed in academic systems, frequently most heavily impacting the hiring and evaluation of women and other historically marginalized groups. It is therefore imperative to continually reexamine the metrics used in hiring and evaluation of research. Conferences, journals, and hiring panels should evaluate applicants through blinded and transparent processes to reduce bias and increase female representation. Institutions should also accept alternative letters of recommendation such as peer letters, mentee letters, or surveys that quantify skills. Furthermore, practices such as cluster hiring can reduce isolation among underrepresented individuals and increase interdisciplinary research. Hiring decisions and negotiations should involve a diverse group of people as well as input from human resources experts with training in bias mitigation in order to ensure that such decisions are handled fairly, sensitively, and equitably. Evaluation in tenure, promotion, and hiring decisions should also be adapted to account for life situations such as pregnancy, parenthood, and family care that disproportionately fall on women, as well as gender-identity-related factors: for example, medical transition for trans individuals can be a time-intensive process that can affect traditional professional timelines.

### 6.5. Provide transparency in financial and career development decisions

Paper authorship, salary negotiations, and funding provisions are all processes that currently operate in an opaque, and therefore inequitable, fashion. To mitigate this, institutions must offer support by illuminating the “hidden curriculum,” improving access to career resources, and offering training in areas such as negotiation techniques, networking, and navigating common career hurdles. To ensure transparency and equity in paper authorship, researchers must have open discussions about authorship and the tasks that contribute to it starting from the beginning of the research process and throughout paper preparation. During hiring, institutions must be transparent to ensure that women know how their offers for salary, start-up package, lab space, etc., compare to other hires.

### 6.6. Provide comprehensive family support

Universities should improve parental benefits at all career stages, since women are disproportionately pushed out of academic career paths due to insufficient support for new parents. Additionally, to reduce the disproportionate burden on women, institutions should make parental and family benefits standard for all new parents, regardless of gender, and ensure that parental leave times are sufficient for the well-being of the infant and family (Earle and Heymann, 2017). The timing and duration of parental leave may still have a negative impact on women’s careers and this must be recognized when evaluating productivity. Funding institutions like the NSF and NIH should recommend increases in post-doctoral salaries so that researchers with young families are better able to support themselves and their families. Moreover, onsite access to child and elder care should be available and affordable for all students and employees. Since many underutilize these benefits for fear of falling behind or retribution, institutions must provide safeguards such as allowing penalty-free family leave, increasing tenure clocks, and developing part-time tenure options.

### 6.7. Reduce the disproportionate burden of non-promotable work

Marginalized academics cannot be made responsible for the majority of critical non-promotable work and departmental service and outreach. Institutions can address this in a few ways. Firstly, by fully acknowledging the importance of these tasks and making them promotable, titled, and compensated, institutions can ensure that this work does not hinder people’s career path. Furthermore, departments can put systems in place to equitably allocate tasks rather than relying on individuals to volunteer. Neural engineers work at the intersection of multiple fields and may therefore experience even broader requests for service, making this a critical priority to ensure that women are not carrying a disproportionate service load.

### 6.8. Ensure that academia is a safe environment

Women must feel welcome and comfortable expressing scientific ideas and asking questions to develop as scientists. No environment is perfect; therefore, it must be clear to all members that the environment is receptive to changes and that those in power will advocate on behalf of historically marginalized groups. Moreover, the environment needs to be welcoming and safe for all members. Institutions should have zero-tolerance policies for sexual harassment and discrimination of any kind. Systems must be put in place to ensure real accountability for perpetrators and support for those affected. Regular evaluations to identify and reduce biases and oversights are critical. Leaders and advisors should also seek out and recruit diverse team members. Diverse teams challenge prevailing perceptions of leadership, and enable innovation driven by distinct perspectives.

## 7. Conclusion

Numerous obstacles contribute to women leaving academia and neural engineering which cannot be remedied without the combined efforts of people from all backgrounds. In this paper, we present many of the barriers to women’s career success in the field as well as a set of recommendations to address these concerns. These include the importance of facilitating a sense of belonging, providing structure for mentoring and networking, designing policies that promote work-life balance, and ensuring that women receive fair credit and compensation for their work. Addressing these barriers will improve the experience of women in neural engineering and increase equity in the field. With career success comes a responsibility to help others overcome obstacles: in short, it is necessary to continue lifting as you climb.

## Author contributions

MJ, JM, AD, JF, EG, and AH performed the interviews. AD and EG collected and compiled the historical data. AD and MJ created the figures. MJ performed the references check. All authors wrote the interview questions, performed literature reviews, wrote sections of the manuscript, and approved the manuscript prior to submission.
