# Supplementary material for: Lifting as we climb: Experiences and recommendations from women in neural engineering
Source: Front Neurosci. 2023 Mar 9;17:1104419. doi: 10.3389/fnins.2023.1104419 (PMC10033556; doi:10.3389/fnins.2023.1104419)
Supplement: Supplementary file 1 [file Data_Sheet_1.PDF]

Interview Questions

Personal experience:
What is the elevator pitch for your research? What was your path to get to your current position?

Did you feel supported in your identity when you were a trainee?

What are the biggest barriers you have faced as a woman (/minoritized person) in STEM?

What everyday strategies do you use in STEM spaces to ensure that your voice is heard and to make space for other minoritized people?

Initiative/support available in neural engineering:
Tell us about any of your initiatives, both formal and informal, to support women. What approaches do you take to uplifting women in neural engineering?

In what ways do you work to uplift other minoritized people in the field?

Quantify: How large is your initiative/what presentations have you done/impact have you had?

Resources:
Are there other initiatives or resources that you think are important for supporting women in neural engineering?

Are there any other resources you're aware of that you think are particularly useful for other women (at any stage of their careers) to know about?

If you had infinite time and money, what resources, policies, funding, or other interventions would you want to institute to support women and minoritized individuals?

Additional questions for Dr. Erika Ross

Personal experience:
Was there a particular reason that decided to leave academia for industry, especially since you were on the tenure track?

What have been the most similar and most different aspects of working in industry compared to academia?

Do you find that support for women in neural engineering is greater or worse in industry compared to academia?

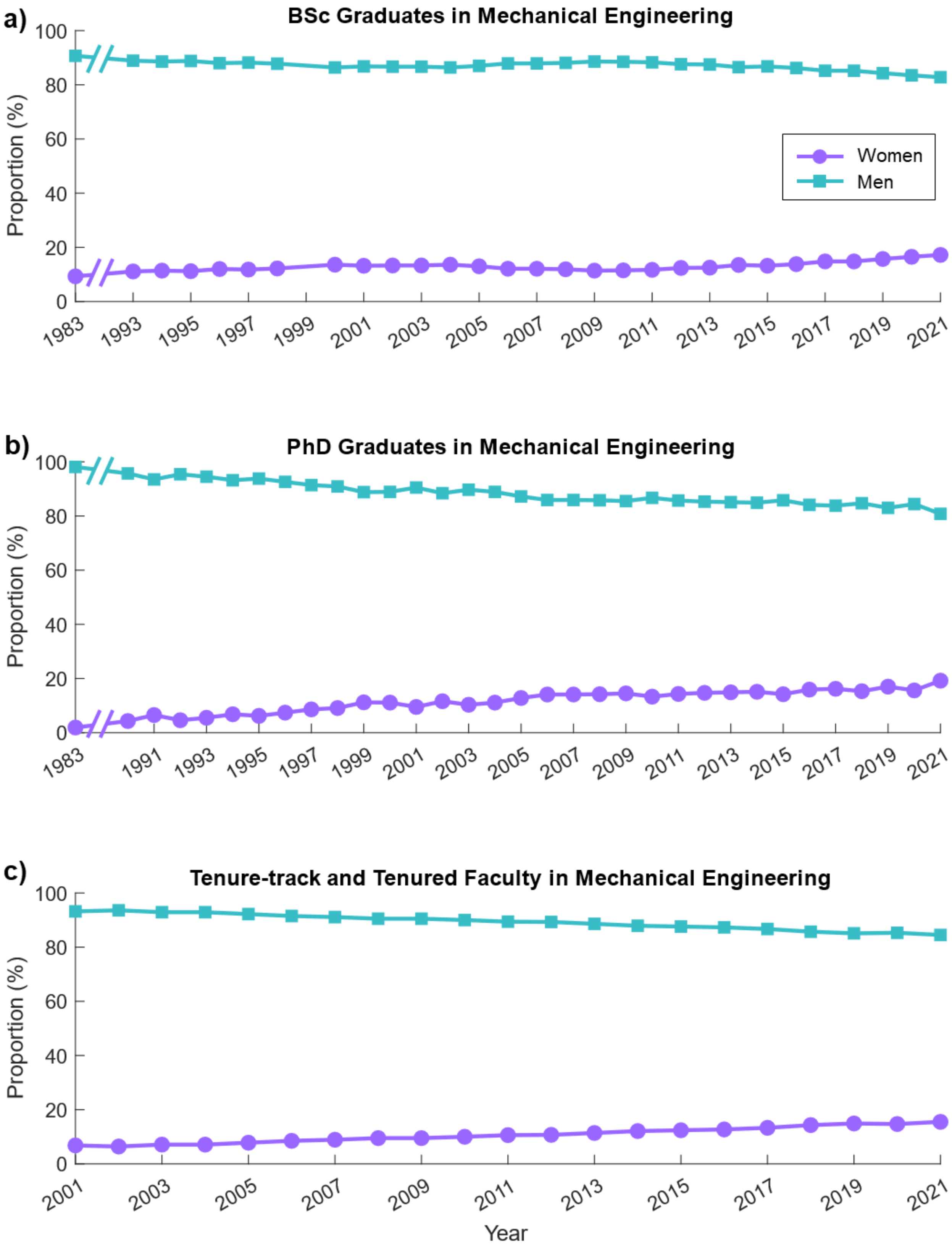

Supplementary Figure 1. Proportion of women in mechanical engineering over time. The proportion of women in mechanical engineering graduating with a bachelor's degree (a), doctorate degree (b) or who are faculty (c) over time. Data were collected from annual and biannual reports from the American Society for Engineering Education and the National Science Foundation. The dashed lines indicate a large gap in data availability.

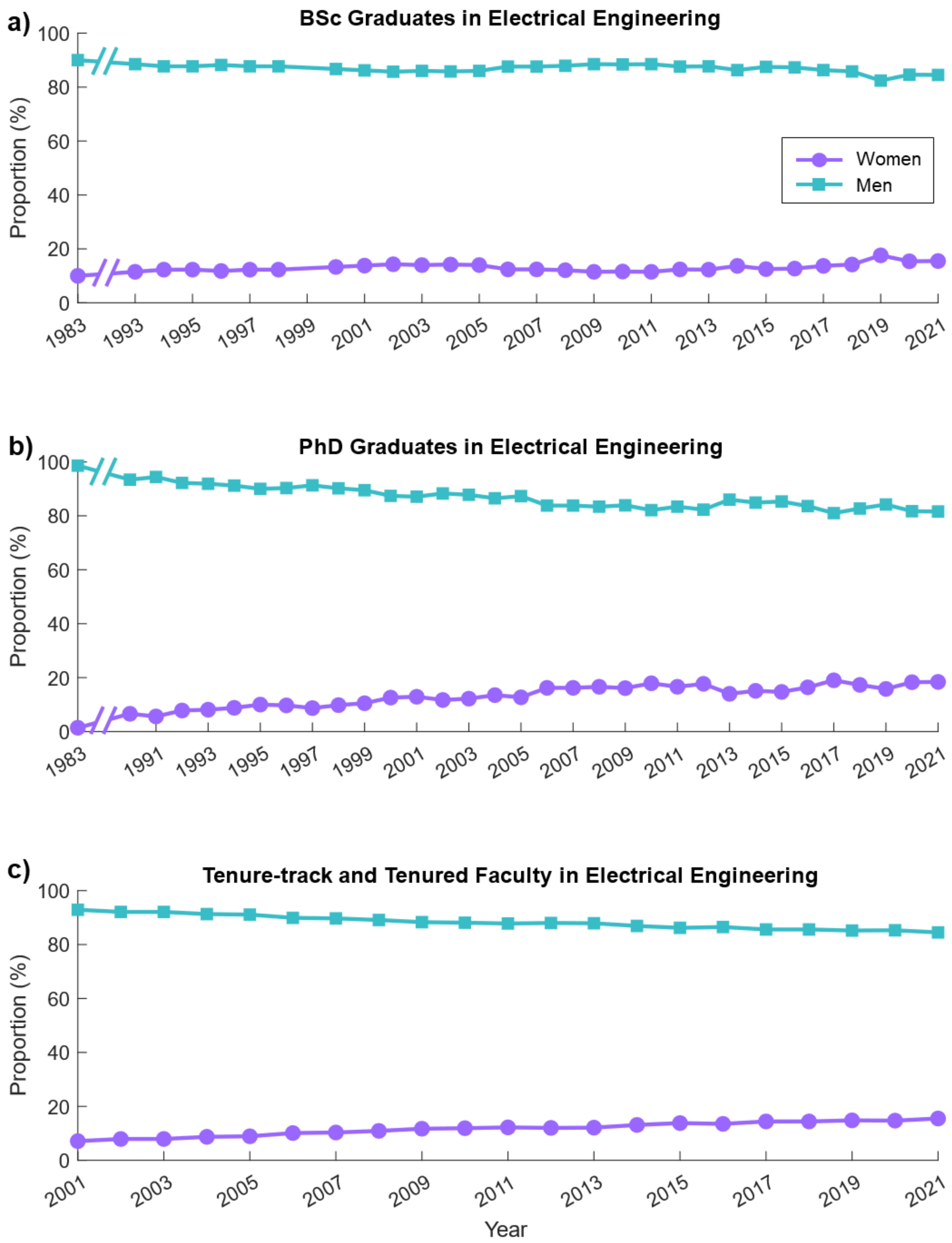

Supplementary Figure 2. Proportion of women in electrical engineering over time. The proportion of women in electrical engineering graduating with a bachelor's degree (a), doctorate degree (b) or who are faculty (c) over time. Data were collected from annual and biannual reports from the American Society for Engineering Education and the National Science Foundation. The dashed lines indicate a large gap in data availability.

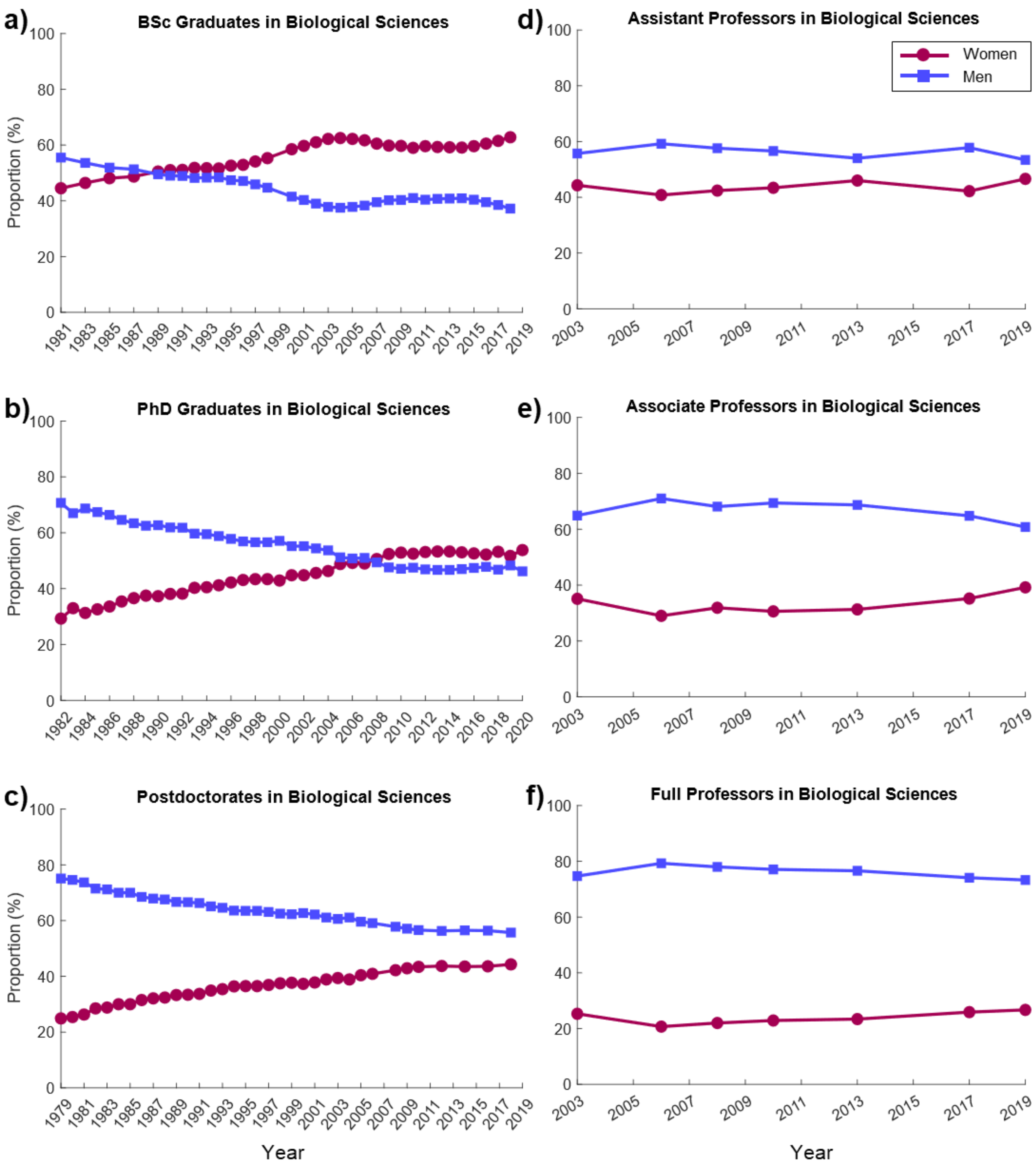

Supplementary Figure 3. Proportion of women in biological sciences over time. The proportion of women in biological sciences graduating with a bachelor's degree (a), doctorate degree (b) or who are a postdoctoral fellow (c), assistant professor (d), associate professor (e), or full professor (f) over time. Data were collected from biannual reports from the National Science Foundation.
