# Supplementary material for: Lifting as we climb: Experiences and recommendations from women in neural engineering
Source: Front Neurosci. 2023 Mar 9;17:1104419. doi: 10.3389/fnins.2023.1104419 (PMC10033556; doi:10.3389/fnins.2023.1104419)
Supplement: Supplementary file 2 [file Table_1.docx]

**Supplementary Table 1.** Informational materials: Funding and resources.

| **Funding Opportunities** |
| --- |
| American Association of University Women AAUW *https://www.aauw.org/resources/programs/fellowships-grants/* |
| L’Oréal Fund for Women *https://www.loreal.com/en/news/commitments/loreal-fund-for-women/* |
| National Science Foundation (NSF) and the Kaleta A. Doolin Foundation Partnership for Gender Diversity *https://www.nsf.gov/news/news_summ.jsp?cntn_id=304777&org=GEO* |
| Nature Awards: Inspiring Women in Science *https://www.nature.com/immersive/inspiringwomeninscience/index.html* |
| NSF ADVANCE: Organizational Change for Gender Equity in STEM Academic Professions *https://beta.nsf.gov/funding/opportunities/advance-organizational-change-gender-equity-stem-academic-professions-advance* |
| Training in Grantsmanship for Rehabilitation Research (TIGRR) |
| **Meetings/Workshops** |
| Big 10 Women's Workshop (BTWW) |
| Center for Neurotechnology (CNT) at the University of Washington Neuroscience for Neurodiverse Learners *https://www.washington.edu/doit/programs/nnl* |
| IEEE Women in Engineering International Leadership Conference *https://ieee-wie-ilc.org/* |
| SACNAS National Diversity in STEM Conference *https://www.sacnas.org/conference* |
| **Organizations** |
| IEEE Women in Engineering (WIE): *https://www.ieee.org/membership/women/index.html*  National Society of Black Engineers (NSBE): *https://www.nsbe.org/*  Black in Neuro: *https://blackinneuro.com/*  Society of Hispanic Professional Engineers (SHPE): *https://shpe.org/*  Society of Women Engineers (SWE): *https://swe.org/*  Women In Neural Engineering (WINE) Forum: *https://womeninneuralengineering.org/*  Brain Computer Interface (BCI) Society: *https://bcisociety.org* |
| **Citation checkers** |
| cleanBib *https://github.com/dalejn/cleanBib* |
| Citation Transparency: Chrome Extension *https://chrome.google.com/webstore/detail/citation-transparency/cepnbdbhabaljgecaddglhhcgajphbcf* |
| GCBI-alyzer: A Gender Citation Balance Index Tool *https://postlab.psych.wisc.edu/gcbialyzer/* |
