# Supplementary material for: Lifting as we climb: Experiences and recommendations from women in neural engineering
Source: Front Neurosci. 2023 Mar 9;17:1104419. doi: 10.3389/fnins.2023.1104419 (PMC10033556; doi:10.3389/fnins.2023.1104419)
Supplement: Supplementary file 3 [file Table_2.docx]

**Supplementary Table 2.** Informational materials for women and mentors of women: Multimedia.

| **Books** | | |
| --- | --- | --- |
| Algorithms of Oppression by *Safiya Noble* | | |
| Blackballed: The Black and White Politics of Race on America's Campuses by *Lawrence Ross* | | |
| Different: Gender Through the Eyes of a Primatologist by *Frans de Waal* | | |
| How to Be an Antiracist by *Ibram X. Kendi* | | |
| Inferior: How Science Got Women Wrong and the New Research That's Rewriting the Story  by *Angela Saini* | | |
| Invisible Women: Data Bias in a World Designed for Men by *Caroline Criado-Perez* | | |
| Mediocre: The Dangerous Legacy of White Male America by *Ijeoma Oluo* | | |
| Our Doctoral Journey: A collection of Black Women's Experiences by *Nicole A. Telfer* | | |
| The No Club: Putting a Stop to Women's Dead-End Work by *Linda Babcock, Brenda Peyser, Lise Vesterlund and Laurie Weingart* | | |
| The Outer Circle: Women in the Scientific Community by *Harriet Zuckerman, Jonathan R. Cole, and John T. Bruer*  The Autobiography of a Transgender Scientist by *Ben Barres* | | |
| **Documentaries** | **Podcasts** | |
| 2017 BMES Diversity Award Lecture: Manu Platt  *https://www.youtube.com/watch?v=s4UKk0zgWi4*  Diversity Up *https://www.youtube.com/channel/UCFjihx66_LbtX-KkFGGnchQ*  Ms. Scientist documentary by Brandy Yanchyk *https://brandyyanchyk.com/ms-scientist*  Picture a Scientist Documentary *https://www.pictureascientist.com/*  Wyatt Cenac’s Problem Areas *HBO Documentary* | Code Switch *https://www.npr.org/podcasts/510312/codeswitch* | |
|  | Lost Women of Science *https://www.scientificamerican.com/author/the-lost-women-of-science-initiative/* | |
|  | Recovering Academic: There is Sunshine Outside the Ivory Tower  *https://recoveringacademic.net/category/uncategorized/* | |
|  | You’re Wrong About   *https://yourewrongabout.com/* | |
|  |  | |
| **Websites** | | |
| Inequality Stories in STEM *https://www.inequalitystoriesinstem.org/* | | |
| Stories of WiN (Women in Neuroscience) *https://www.storiesofwin.org* | | |
| SfN History of Neuroscience *https://www.sfn.org/about/history-of-neuroscience* | | |
| 500 Queer Scientists Campaign [*https://500queerscientists.com*](https://500queerscientists.com)  Anne’s List  *https://anneslist.net/* | | |
|  | | |
|  | | |
| **Twitter** | |  |
| 500 Women Scientists: *@500womensci* | |  |
| Academic Mom, PhD: @*Momademia* | |  |
| BiasWatchNeuro: *@BiasWatchNeuro* | |  |
| Black in BME: *@BlackInBME* | |  |
| Queer in Neuro: *@QueerInNeuro* | |  |
| Women In Neural Engineering Forum: *@WINE_Forum* | |  |
| Women in Neuroscience: *@Women_inNeuro* | |  |
| Black In Neuro: *@BlackInNeuro* | |  |
| BlackAFinSTEM: *@BlackAFinSTEM*  LatinXinBME: *@LatinXinBME* | |  |
| Repository for Women in Neuroscience: *@WINRePo1* | |  |
| storiesofWiN: *@storiesofwin* | |  |
| Women's History Month Spotlighting Neuroscientists by Dr. Amy Orsborn *https://twitter.com/neuroamyo/status/1498674431542972416*  International Women’s Day thread on gender disparity in STEM by Dr. Zoë Ayres *https://twitter.com/ZJAyres/status/1501108708570546180?t=xhrri0FKhHYLrthZoamb_A&s=19* | |  |
